# Genetic Divergence and Isolation of the Green Sea Turtle (*Chelonia mydas*) in the Red Sea

**DOI:** 10.1002/ece3.72046

**Published:** 2025-08-25

**Authors:** K. Scott, S. He, R. S. Hardenstine, L. K. Tanabe, H. Barrios‐Garrido, M. P. Jensen, L. Afiq‐Rosli, N. E. Wildermann, C. M. Duarte, J. E. M. Cochran, M. L. Berumen

**Affiliations:** ^1^ Marine Science Program, Division of Biological and Environmental Science and Engineering King Abdullah University of Science and Technology Thuwal Saudi Arabia; ^2^ KAUST Beacon Development, KAUST National Transformation Institute, Innovation Cluster, 4700 King Abdullah University of Science and Technology Thuwal Saudi Arabia; ^3^ Asian School of the Environment Nanyang Technological University Singapore City Singapore; ^4^ World Wide Fund for Nature, Healthy Land and Seascapes Brisbane Queensland Australia; ^5^ Department of Chemistry and Bioscience Aalborg University Aalborg Denmark

**Keywords:** *Chelonia mydas*, mtDNA, population genetics, Red Sea

## Abstract

The green sea turtle *Chelonia mydas* is a wide‐ranging marine reptile, inhabiting all the world's tropical and warm‐temperate seas. This global distribution makes delineating population boundaries challenging, and molecular tools like genetic markers are often required to define these limits. The Red Sea hosts ~1500 nesting green turtles, but research in the region is limited. The Red Sea may foster genetically distinct populations due to geographic barriers and extreme environmental conditions, as observed in other Red Sea taxa. In the present study, we sampled 193 nesting females and hatchlings from five rookeries, which support ~550 nesting females per year, on the Saudi Arabian coast of the Red Sea. We aimed to determine the genetic diversity within the basin and connectivity around the Arabian Peninsula and to the wider Indian Ocean. We analysed 710 bp of the mtDNA d‐loop from five Red Sea rookeries. Five haplotypes, grouped into two highly divergent haplogroups, were identified. Haplogroup one comprised 85% of our sample library and four haplotypes (CmP71.1–CmP71.4), three of which were previously unreported, while haplogroup two was much less prevalent (15% of the sample library) and consisted of a single novel haplotype (CmP62.2) more closely related to Arabian Gulf haplotypes. Most Red Sea rookeries shared haplotypes, but we observed a slight north‐to‐south gradient due to the increasing prevalence of CmP62.2. Pairwise *F*_st_ differences showed high genetic differentiation between the Ras al Baridi and the Al Wejh rookeries, indicating restricted gene flow between two major nesting sites and highlighting the need for separate conservation management strategies within the basin. We suggest that the isolation of the Red Sea and past paleo and climatic events have led to the divergence of three endemic haplotypes and possibly an evolutionary adaptation to the elevated temperature within the basin, which warrants further study.

## Graphical abstract

The green sea turtle population in the Red Sea, comprising around 5000 nesting individuals, shows genetic differentiation across five rookeries sampled on the Saudi Arabian coast. Analysis of mitochondrial DNA revealed five haplotypes grouped into two distinct haplogroups, indicating limited gene flow between rookeries and supporting the possibility of isolated populations. The findings suggest that geographic isolation, climatic factors and past evolutionary events may have contributed to this genetic divergence, emphasising the need for tailored conservation strategies in the region.

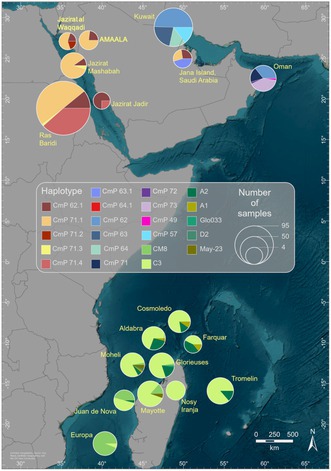

## 1 Introduction

Green sea turtles ( *Chelonia mydas* , Linnaeus, 1758) inhabit tropical and subtropical latitudes (Lutz et al. 2002). Due to life history traits such as long lifespans and their highly migratory nature (Lohmann et al. 1999), sea turtles have increased susceptibility to threats (Wallace et al. 2011), such as fisheries bycatch (López‐Mendilaharsu et al. 2020), boat strikes (Shimada et al. 2017), direct take (Joseph et al. 2019; Pheasey et al. 2021) and coastal development (Goldberg et al. 2015). This has led to the historic depletion of some stocks, resulting in their classification as globally Endangered on the International Union for the Conservation of Nature (IUCN) Red List (Seminoff 2023), although there is encouraging evidence of recovery (Chaloupka et al. 2008; Mazaris et al. 2017; Weber et al. 2014). Moreover, their dispersive lifestyle (Luschi et al. 2003), combined with a lengthy maturation period of over 40 years for some populations (Goshe et al. 2010), means that individuals can have multiple critical habitats, often thousands of kilometers apart (Pike 2013). This makes population dynamics difficult to study at regional scales (Bowen and Karl 2007), and hinders potential conservation efforts, especially for green sea turtle populations in understudied regions (Al Ameri et al. 2022), such as the Red Sea.

The Red Sea is a semi‐enclosed oligotrophic ocean basin separated from the Indian Ocean by a shallow and narrow (~30 km) opening called the Strait of Bab‐el‐Mandeb and the Mediterranean Sea by the Gulf of Suez (Rasul and Stewart 2015). Five of the seven extant sea turtle species inhabit the Red Sea, with the green and hawksbill turtle ( *Eretmochelys imbricata* , Linnaeus, 1766) nesting most frequently. The green sea turtle nests along the entirety of the Saudi Arabian coast of the Red Sea, on beaches and offshore islands (Shimada et al. 2021). Current population estimates are around 1500 turtles nesting per year in the entire Red Sea (Hanafy 2012). The major and most studied rookeries are concentrated in the northern Red Sea, the largest being Ras al Baridi, hosting approximately 250 to 300 nesting females a year (Miller 1989; Shimada et al. 2021). Although all countries bordering the Red Sea protect sea turtles through national and international agreements (Mancini et al. 2015), sea turtle research and conservation efforts are still in their infancy in this basin, lacking basic information on foraging and breeding populations.

Molecular techniques are essential for defining distinct breeding populations, which is critical for the effective conservation of highly migratory species (Hardenstine et al. 2022), like marine turtles (Palumbi 2003). Population genetics offers a means to understand the ecology of highly migratory species by assessing stock structure and connectivity (Avise 1992; Bowen and Karl 2007; Jensen, FitzSimmons, et al. 2019). Sea turtles exhibit nest site fidelity, with females returning to beaches near their hatching sites to lay their eggs. This behaviour results in localised breeding populations and genetically distinct groups, with genetic stocks structured matrilineally (Bowen et al. 1992; Bowen and Karl 2007; Jensen, FitzSimmons, et al. 2019; Ng et al. 2024). Therefore, many studies use mitochondrial DNA (mtDNA), inherited from the mother, to detect boundaries in nesting populations (Lahanas et al. 1994; Formia et al. 2006; Jensen, FitzSimmons, et al. 2019). Mitochondrial DNA has a fast evolution rate and therefore serves as an effective marker for studying population structure, genetic diversity and phylogeography, enabling the identification of distinct groups within and between rookeries (Naro‐Maciel et al. 2008; Leroux et al. 2012; Yang et al. 2015; Vargas et al. 2016). Quantifying the genetic diversity within these populations can provide insights into the adaptive capacity of Red Sea green turtles, which is particularly pertinent for sea turtle species and their response to climate change (Ekanayake et al. 2017). Understanding the population structure and genetic diversity of green sea turtle populations will help inform management decisions by identifying demographically independent breeding populations locally and support the designation of regional management units (RMUs) more broadly (Wallace et al. 2010, 2023). To date, three studies with small sample sizes have been published on green sea turtle genetics in the Red Sea (*n* = 4 (Tikochinski et al. 2012); *n* = 16 (Jensen, Miller, et al. 2019); *n* = 17 (Naguib et al. 2022)). Among these, only Jensen, Miller, et al. (2019) focused on investigating population structure and connectivity. Enhancing our understanding of sea turtle genetics is essential given the escalating pressures on this species due to extensive development along the Red Sea coastline, where several large‐scale gigaprojects, such as the Red Sea Project, AMAALA and NEOM (Robitzch et al. 2023), are being developed in areas that include established sea turtle rookeries (Shimada et al. 2021).

This study aims to build on previous works by examining the mtDNA control region polymorphism, incorporating five rookeries, four of which have not been included in previous studies and a higher sample size to refine population delineation within the Red Sea. We analysed tissue samples from 193 individuals encompassing five rookeries (Jazirat Mashabah, Ras al Baridi, Jazirat Jadir, Jazirat Waqqadi and AMAALA), to identify haplotypes, determine genetic diversity and connectivity with other populations around the Arabian Peninsula. Identifying breeding stocks and estimates of genetic diversity within the Red Sea will aid in conservation planning and increase understanding of the adaptive potential of this species in a warming environment.

## 2 Materials and Methods

### 2.1 Study Sites

**FIGURE 1:**
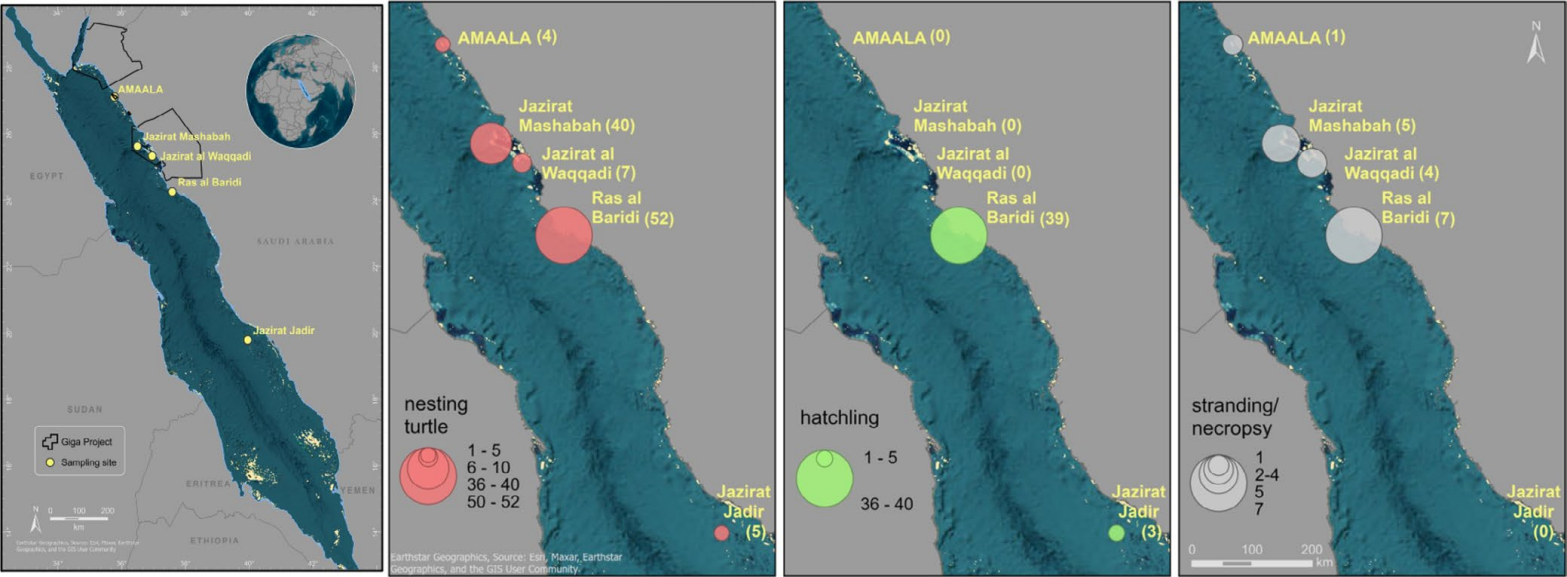
Sampling rookeries for green sea turtles are distributed along the Saudi Arabian coast of the Red Sea, within key development regions: AMAALA, Jazirat Mashabah, Jazirat al Waqqadi, Ras al Baridi and Jazirat Jadir. The nesting turtle, hatchling and strandings/necropsy sampling sizes are linked with circle sizes and scaled as presented in the legends. The Giga project areas are delimited with a black line.

Tissue samples were collected between 2019 and 2024 along the Saudi Arabian coast of the Red Sea, spanning 28° to 19° latitude (Figure 1). We sampled five known green sea turtle rookeries, including four islands—AMAALA (27.10529°, 35.77037°), Jazirat Mashabah (25.62484°, 36.49650°), Jazirat Waqqadi (25.33219°, 36.95913°) and Jazirat Jadir (19.78931°, 39.95355°), and one mainland site, Ras al Baridi (24.25009°, 37.58876°). Jazirat Mashabah and Jazirat Waqqadi are part of the Al Wejh lagoon, a coastal system in the northern Red Sea that contains 92 islands and serves as an important marine habitat (Chalastani et al. 2020). This region is also undergoing large‐scale coastal development as part of the Red Sea Project. Jazirat Mashabah, also known as Breem Island, is situated on the northwest outer edge of the lagoon and supports an estimated 150 nesting females per year. Jazirat Waqqadi, located just south of the lagoon, hosts a smaller rookery with approximately 50 nesting individuals per year (Shimada et al. 2021).

Ras al Baridi is the largest known green turtle rookery in the Red Sea, with 250 to 300 nesting females per year (Al‐Merghani et al. 2000; Shimada et al. 2021). Nesting numbers at this site, as well as at Jazirat Mashabah, have increased over the past three decades (Shimada et al. 2021). Jazirat Jadir, located 50 km offshore from the coastal city of Al Lith in the southern Red Sea, is a smaller rookery hosting around 50 nesting individuals annually (Shimada et al. 2021).

The AMAALA gigaproject, situated in the northern Red Sea, represents a smaller rookery with fewer than 50 nesting females per year (Shimada et al. 2021). This site has been selected for one of the kingdom's large‐scale ecotourism projects (Robitzch et al. 2023).

### 2.2 Sample Collection

Nesting beaches were patrolled at night along the high tide line to locate fresh sea turtle tracks. All fresh tracks were followed to locate the nesting female. Females were approached after oviposition was complete to minimise disturbance. Before tissue collection, the sampling site was disinfected using 70% ethanol. Samples were taken from the trailing axial edge of one of the front flippers using forceps and a sterile stainless‐steel disposable scalpel (Dutton 1996). To prevent duplicating samples, individuals were flipper‐tagged with a unique code. Evidence suggests that tags lasted multiple nesting seasons, with one individual tagged on Jazirat Mashabah in 2021 resighted in 2023 with the flipper tag still legible and intact. Previous research has shown tag retention in the Red Sea for up to 32 years (Alatawi 2024). When flipper‐tagging was not possible, only individuals that nested were sampled, ensuring that the sampling campaign duration remained shorter than the inter‐nesting period (~10 days) to avoid resampling.

For necropsy specimens (the individual is deceased), tissues were collected from the least decomposed area to ensure sample integrity. Live hatchling samples were obtained using a 4 mm biopsy punch to extract a small core from one of the marginal scutes (González‐Garza et al. 2016). Hatchlings found dead onshore were sampled using a scalpel and forceps. After sampling, all tissue was preserved in a 96% ethanol solution and kept in a −20°C freezer until DNA extraction.

Between 2019 and 2024, a total of 232 green sea turtle individuals were sampled. The majority (*n* = 144) were collected from the Ras Al Baridi rookery, including 41 hatchlings found opportunistically on the nesting beach. At Jazirat Mashabah, samples were collected from 41 individuals, mainly nesting females (*n* = 39), along with two necropsy samples. From the Jazirat Waqqadi rookery, we collected samples from 12 individuals, mainly nesting females (*n* = 10), and two necropsy samples. From Jazirat Jadir, samples were collected from nesting females (*n* = 4) and hatchlings (*n* = 3). At the AMAALA rookery, we obtained four samples from nesting females and one hatchling sample. The remainder of the samples (*n* = 23) were collected at various sites along the Saudi Arabian coast, including stranded turtles (the individual is still alive) (*n* = 3), necropsies (*n* = 11) and foraging turtles (*n* = 1). Herein, we refer to these as ‘unknown’ as their natal rookery was not identified.

All sampling was conducted under appropriate animal ethics protocols, under KAUST's Institutional Animal Ethics Care and Use Committee (IACUC) protocols 19IACUC07 and 18IACUC11. Additionally, all field research was conducted under permit RS‐22‐00005, issued by Saudi Arabia's National Centre for Wildlife (NCW) and the authorisation number HAP‐02‐J‐42 provided by the National Committee of Bioethics.

### 2.3 Laboratory Analysis

DNA extraction was performed by tissue digestion in proteinase K for all 232 samples, using a DNEasy Blood and Tissue Kit (Qiagen, Hilden, Germany) following the manufacturer's protocol. Extracted DNA concentrations were quantified using a Qubit Fluorometric Quantification System (Thermofisher Scientific, Waltham, MA, USA). We obtained a DNA concentration of > 1 ng/mL for 196 samples, which were used in further analysis.

An ~800 base pair (bp) fragment of the mtDNA control region was amplified using polymerase chain reaction (PCR) for all samples (*n* = 196) using a SimpliAmp thermocycler (Applied Biosystems, Foster City, CA). The reaction was carried out on 15 μL total volume using primers, 0.75 μL of forward primers LCM15382 (5′‐GCTTAACCCTAAAGCATTGG‐3′) or LTei9 (5′‐GGGAATAATCAAAAGAGAAGG‐3′) and 0.75 μL of reverse primer H950 (5′‐GTCTCGGATTTAGGGGTTTG‐3′) (Abreu‐Grobois et al. 2006) and 7.5 μL mastermix (Qiagen, Hilden, Germany).

The thermocycler started with an initial phase of 95°C for 15 min, followed by 35 cycles of 94°C for 30 s, 50°C for 1 min, 72°C for 1 min and with a final single extension of 72°C for 10 min. PCR products were visualised on a QIAxcel DNA Screening Kit (Qiagen) or by gel electrophoresis at 1.5% gel (1.5 g agarose in 100 mL 1× TAE buffer). All successfully amplified DNA was purified by adding 2 μL of ExoProStar (GE Healthcare, Chicago, IL, USA) to a 5 μL aliquot of the PCR product before running a single thermal cycle of 37°C for 15 min and 80°C for 15 min. Sanger sequencing was conducted using the 3730xl DNA Analyser (Applied Biosystems, Foster City, CA, USA) at the King Abdullah University of Science and Technology, BioScience Core Labs.

### 2.4 Data Analysis

#### 2.4.1 Sequence Processing and Alignment

All successfully amplified sequences (*n* = 193, nesting, hatchling and unknown samples) were initially ~800 bp in length; however, only 670 bp were used for analysis due to low sequence quality at the amplicon ends. Sequences were trimmed to 710 bp to maintain accuracy and consistency across all samples. Alignment was conducted in Geneious Prime (Version 2023.2.1, Biomatters Ltd., Auckland, New Zealand) using the ‘Global alignment with free end gaps’ algorithm, with the cost matrix set to ‘Identity (1.0/0.0)’. Sequences were grouped into haplotypes using DNAsp v6.12.03 (Librado and Rozas 2009) and identified using a search on the ShellBank marine turtle genetic database (www.shellbankproject.org) as well as a Blast search on GenBank (https://blast.ncbi.nlm.nih.gov/). Newly identified haplotypes were given unique designations, while previously reported haplotypes followed established nomenclature. A representative sequence for each haplotype was deposited in GenBank (accession numbers PQ336929–PQ336933).

#### 2.4.2 Network Construction

To assess genetic relationships and population structure, we constructed two haplotype networks using mitochondrial DNA sequences. The first network focused on the Red Sea samples using a longer 710 bp fragment length to enhance genetic resolution within our dataset. To provide a wider regional context, additional sequences were downloaded from GenBank, including datasets from Kuwait (Al‐Mohanna et al. 2014), Saudi Arabia (Jana Island, Jensen, Miller, et al. 2019) and Oman (Reece et al. 2016). We truncated our sequences to create a second network based on a shorter 384 bp fragment length, facilitating comparative analysis for the entire Arabian Peninsula. Future work should focus on extending the fragment length to increase the resolution of this analysis. A median‐joining haplotype network was constructed in NETWORK v10.2.0.0 (Bandelt et al. 1999) for each dataset to visualise genetic relationships among haplotypes. Insertions and deletions resulted in sequences of various lengths when they were aligned and compared with those obtained from GenBank. Typically, insertion/deletion gaps in an alignment are phylogenetically informative (Pearce 2005) and have rarely been discussed in previous studies (Al‐Mohanna et al. 2014; Phillott and Gamage 2014; Reece et al. 2016; Jensen, Miller, et al. 2019; Jensen, FitzSimmons, et al. 2019), so we included them in our analysis. To facilitate comparison with previous work and to investigate the bioinformatic value of insertion/deletion sites, two files with and without insertion/deletion gaps were generated by DNASP v6 12.03, which were used as input for the construction of haplotype networks (.rdf) in NETWORK v10.2.0.0 (Bandelt et al. 1999) and for population genetic analysis (.nexus) in Arlequin. The gaps considered approach was used in further analysis and presented in the results, but a median‐joining network showing substitutions only is available in Figure S1.

#### 2.4.3 Genetic Diversity and Population Structure Analyses

Genetic diversity indices, including haplotype frequencies, haplotype diversity (*Hd*) and nucleotide diversity (*πd*), were calculated for each rookery. To examine population structure, pairwise comparisons of fixation indices (*F*_st_), based on conventional haplotype frequencies, were conducted, with significance tested using 10,100 permutations and a threshold of *p* < 0.05. Due to their close proximity, Jazirat Mashabah and Jazirat Waqqadi were combined in this analysis, referred to as Al Wejh rookeries in the results. However, a full table of all *F*_st_ values can be found in Table S2. Additionally, a one‐way analysis of molecular variance (AMOVA) was conducted to assess genetic differentiation among rookeries. All statistical analyses were performed using Arlequin v3.5.2.2. Our unknown samples were removed from population structure analysis, as their natal origin was unknown and therefore could not be assigned with certainty to a rookery.

## 3 Results

### 3.1 Red Sea Populations (670 Bp Dataset)

**FIGURE 2:**
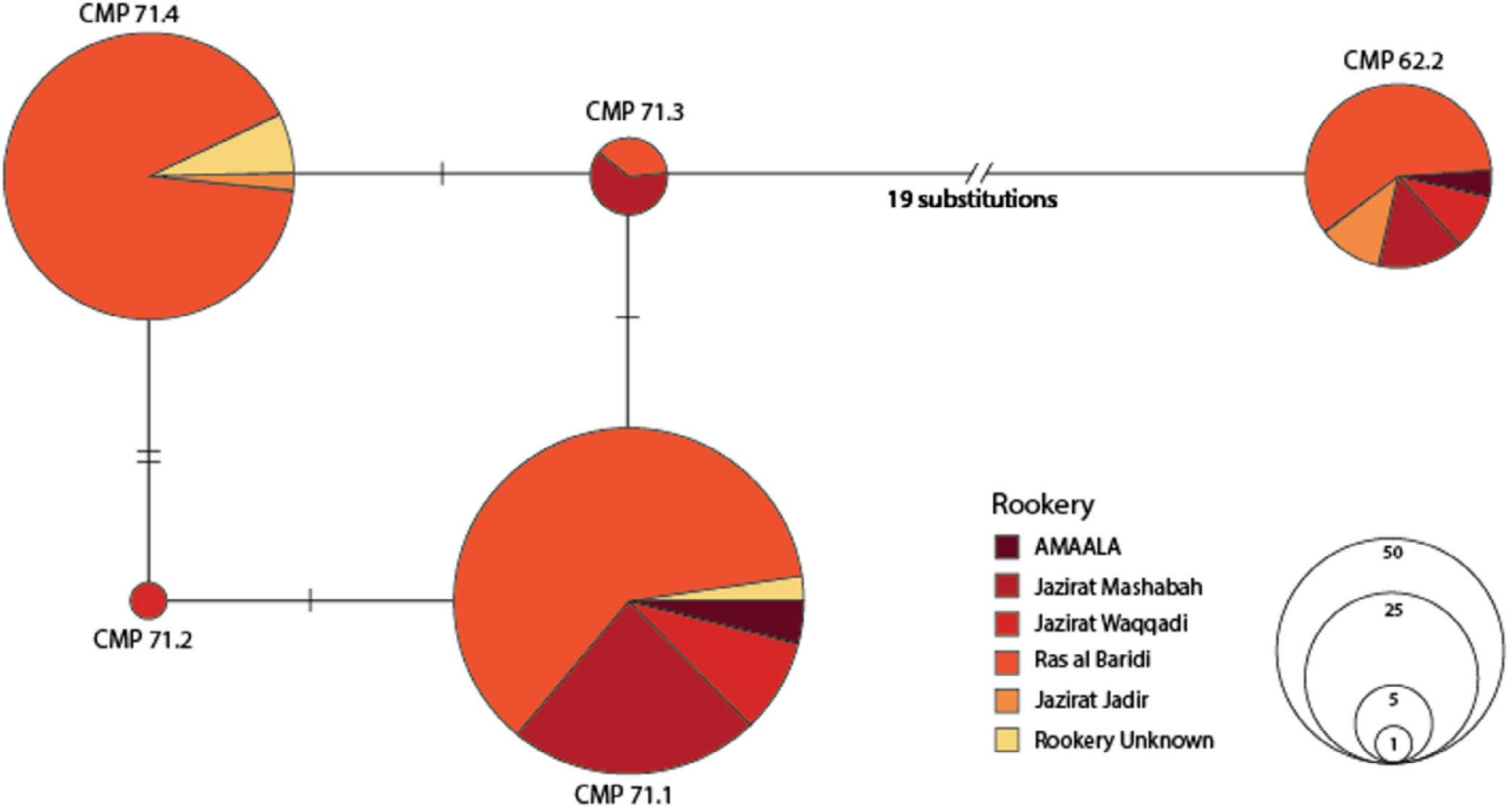
Haplotype network for the green turtle populations based on 670 bp mitochondrial control region fragments, distributed in the Red Sea. The legend indicates where distinct rookery colours are found the haplotype, and the size of the circles is proportional to their total frequency. Each branch connecting different circles represents the mutation steps among the haplotypes, with the black crossbars representing an additional nucleotide substitution, a single deletion/insertion, and the double bars representing greater than one nucleotide substitution. Unknown samples are those with unknown natal origin.

A median‐joining haplotype network constructed using the 670 bp dataset revealed two highly divergent haplogroups, separated by 19 nucleotide substitutions. Haplogroup 1 contained four closely related haplotypes (CmP71.1, CmP71.2, CmP71.3 and CmP71.4), while Haplogroup 2 consisted of a single haplotype, CmP62.2 (Figure 2).

Among the 193 successfully sequenced samples, a total of five distinct haplotypes were identified, four of which had not been previously reported. Haplotypes were assigned using the ShellBank marine turtle genetic database and confirmed through GenBank BLAST searches. Newly identified haplotypes were assigned unique designations, and representative sequences were deposited in GenBank (PQ336929–PQ336933). The most common haplotype, CmP71.1, was found in 90 individuals and had been previously reported in the Red Sea (Jensen, Miller, et al. 2019). The newly identified haplotypes included CmP71.4 (*n* = 69), CmP62.2 (*n* = 28), CmP71.3 (*n* = 5) and CmP71.2 (*n* = 1). The CmP62.2 haplotype was observed at all sampled Red Sea sites but was particularly prevalent among the sample from Jazirat Jadir. The other newly identified haplotypes showed varying distributions, with CmP71.3 primarily found in Ras al Baridi and Jazirat Mashabah, while CmP71.2 was detected in a single individual from Jazirat Waqqadi.

Haplotype and nucleotide diversity varied among Red Sea rookeries. Ras al Baridi exhibited the highest haplotype diversity (*Hd* = 0.6164), followed by samples of unknown origin (*Hd* = 0.5357). Among the other rookeries, Jazirat Jadir and Jazirat Waqqadi showed moderate haplotype diversity (*Hd* = 0.5000), while Jazirat Mashabah displayed lower haplotype diversity (*Hd* = 0.3770). Nucleotide diversity (*πd*) was highest at Jazirat Jadir (*πd* = 0.1693), significantly greater than any other site. The unknown samples exhibited the second highest nucleotide diversity (*πd* = 0.0669), followed by AMAALA (*πd* = 0.0130) and Jazirat Waqqadi (*πd* = 0.0125) (Table 1).

**TABLE 1:** Genetic diversity indices obtained for green turtle populations of the Red Sea based on the mitochondrial control region, compared with Arabian Gulf, Northwestern Indian Ocean and 10 other rookeries next to Seychelles, Madagascar and the Mozambique Channel.

Rookery	n	Original fragment length (bp)	No. of haplotypes	Haplotypes	Hd	SD	*π*d	SD	References
Jazirat Waqqadi (50)	13	670	3	CmP71.1 (9), CmP71.2 (1), CmP62.2 (2)	0.5000	±0.1364	0.0125	±0.0069	This study
AMAALA (50)	5	670	2	CmP71.1 (4), CmP62.2 (1)	0.4000	±0.2373	0.0130	±0.0084	This study
Jazirat Jadir (50)	4	670	2	CmP62.2 (3), CmP71.4 (1)	0.5000	±0.2562	0.1693	±0.0116	This study
Jazirat Mashabah (150)	32	670	3	CmP71.1 (27), CmP71.3 (1), CmP62.2 (4)	0.3770	±0.0987	0.0073	±0.0040	This study
Ras al Baridi (250–300)	131	670	4	CmP71.1 (63), 71.2 (5), 71.4 (49), CmP62.2 (14)	0.6164	±0.0220	0.0075	±0.0040	This study
Jana Island	11	770	3	CmP62.1 (3), CmP63.1 (5), CmP64.1 (3)	0.709	±0.083	0.003	±0.002	Jensen, Miller, et al. (2019)
Ras al Hadd, Oman	42	384	5	CmP62 (15), CmP71 (8), CmP72 (1), CmP73 (17), CmP49 (1)	0.688	±0.035	0.022	±0.011	Reece et al. (2016)
Qaruh & Umm Al Maradim, Kuwait	97	384	4	CmP62 (47), CmP63 (21), CmP64 (13), CmP57 (14), CmP72 (2)	0.673	±0.034	0.004	±0.003	Al‐Mohanna et al. (2014)
Europa	33	396	2	CM8 (31), C3 (2)	0.1174	—	0.0076	—	Bourjea et al. (2007)
Juan de Nova	20	396	3	CM8 (11), C3 (8), A2 (1)	0.5632	—	0.0360	—	Bourjea et al. (2007)
Nosy Iranja	13	396	1	C3 (13)	0	—	0	—	Bourjea et al. (2007)
Mayotte	41	396	5	CM8 (5), C3 (30), May23 (2), A1 (1), A3 (3)	0.4524	—	0.0231	—	Bourjea et al. (2007)
Moheli	34	396	6	CM8 (1), C3 (27), May23 (2), D2 (1), A1 (1), A2 (2)	0.3708	—	0.0133	—	Bourjea et al. (2007)
Gloerieuses	39	396	3	C3 (31), Glo33 (1), A2 (7)	0.3441	—	0.0168	—	Bourjea et al. (2007)
Cosmoledo	31	396	3	C3 (24), A1 (3), A2 (4)	0.3871	—	0.0210	—	Bourjea et al. (2007)
Aldabra	26	396	3	C3 (18), A1 (1), A2 (7)	0.4646	—	0.0249	—	Bourjea et al. (2007)
Farquar	7	396	3	C3 (3), A1 (1), A2 (7)	0.7143	—	0.0342	—	Bourjea et al. (2007)
Tromelin	44	396	2	C3 (38), A2 (6)	0.2410	—	0.0132	—	Bourjea et al. (2007)

*Note:* The acronyms *n* denotes the number of samples, *Hd* the haplotype diversity and *π* the nucleotide diversity. Fragment lengths were trimmed to 384 bp, the most conservative fragment length, which served as the basis for calculating diversity indices.

### 3.2 Comparative Analysis With Arabian Gulf Populations (384 Bp Dataset)

**FIGURE 3:**
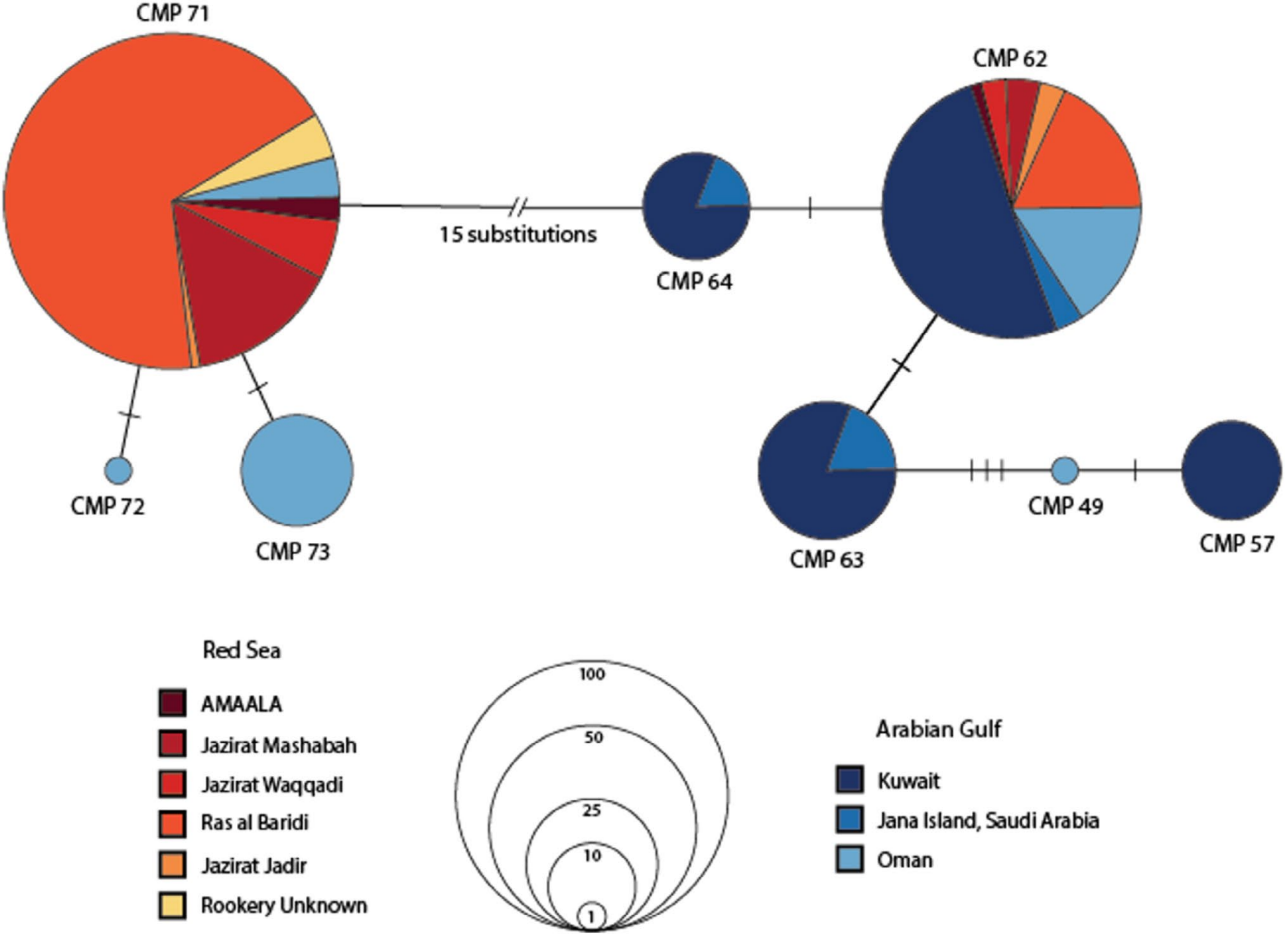
Haplotype network for the green turtle populations based on 384 bp mitochondrial region fragments, distributed on the Arabian Peninsula. The legend indicates where distinct rookery colours are found the haplotype, and the size of the circles is proportional to their total frequency. Each branch connecting different circles represents the mutation steps among the haplotypes, with the black crossbars representing an additional nucleotide substitution, single insertion/deletion (Table 1), and the double bars representing greater than one nucleotide substitution. Unknown samples are those with unknown natal origin.

**FIGURE 4:**
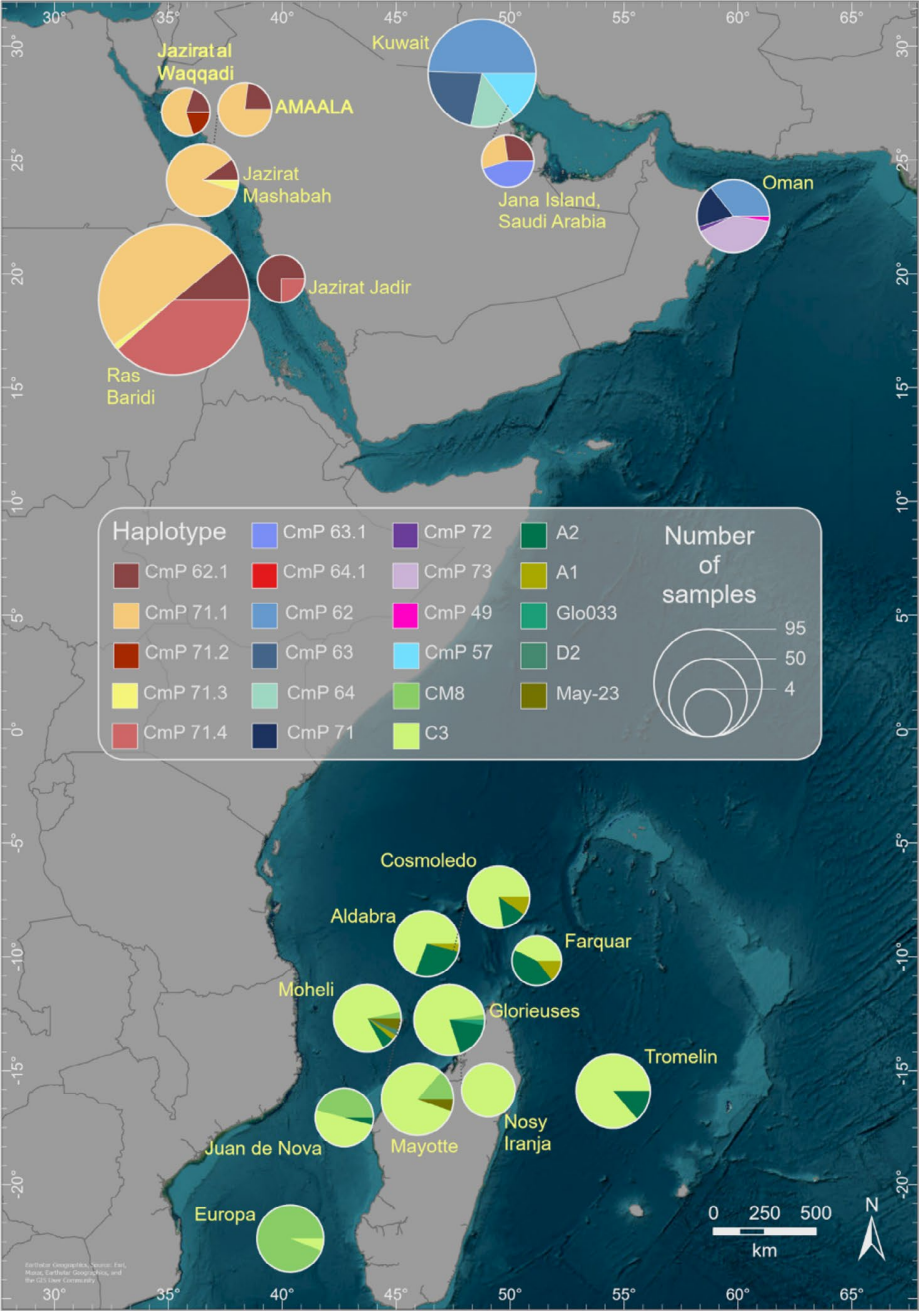
Geographical distribution of haplotype frequencies (pie charts) found in green turtle rookeries of the Red Sea (this study), Arabian Gulf (Al‐Mohanna et al. 2014; Reece et al. 2016; Jensen, Miller, et al. 2019) and 10 other rookeries next to Seychelles, Madagascar and Mozambique Channel (Bourjea et al. 2007). The legend indicates the colours for each haplotype and its respective number of samples. Haplotype frequencies are based on original fragment length.

To compare Red Sea rookeries with Arabian Gulf populations, all sequences were trimmed to 384 bp to allow direct comparison with published datasets from Kuwait, Saudi Arabia (Jana Island) and Oman (Al‐Mohanna et al. 2014; Jensen, Miller, et al. 2019; Jensen, FitzSimmons, et al. 2019; Reece et al. 2016). The 384 bp haplotype network revealed that, similar to the Arabian Gulf and Southwest Indian Ocean, the Red Sea was dominated by a primary haplotype (CmP71), analogous to CmP62 in the Arabian Gulf (Figure 3) and C3 in the Southwest Indian Ocean (Figure 4). However, unlike the Arabian Gulf and Southwest Indian Ocean, which contain multiple unique haplotypes, the Red Sea rookeries displayed less haplotype diversity (only two in the 384 bp network). Jana Island (Saudi Arabia), Oman and Kuwait had a higher number of unique haplotypes compared to the Red Sea.

### 3.3 Population Structure and Genetic Differentiation

To assess genetic structure within the Red Sea and across the Arabian Peninsula, pairwise *F*_st_ comparisons and AMOVA were performed separately for the 670 bp (Red Sea) and 384 bp (Arabian Peninsula) datasets. AMOVA comparing Red Sea versus Arabian Gulf rookeries revealed significant genetic differentiation (*F*_st_ = 0.47609, *p* < 0.001). Pairwise *F*_st_ comparisons showed that both Red Sea rookeries (Ras al Baridi and Al Wejh rookeries) were significantly different from all Arabian Gulf sites (Table 2). Within the Red Sea, pairwise *F*_st_ comparisons indicated significant genetic structuring, particularly between Ras al Baridi and Al Wejh rookeries (*F*_st_ = 0.2789, *p* < 0.01). Due to small sample sizes, Jazirat Jadir and AMAALA were omitted from pairwise *F*_st_ analysis, but their genetic relationships to other sites are presented in Figure S2.

**TABLE 2:** Pairwise *F*_st_ values comparing green turtle rookeries of the Arabian Peninsula (this study; Al‐Mohanna et al. 2014; Reece et al. 2016; Jensen, Miller, et al. 2019) for 384 bp mitochondrial control region.

	Ras al Baridi (*n* = 131)	Al Wejh (*n* = 45)	Oman (*n* = 42)	Kuwait (*n* = 95)	Jana Island (*n* = 11)
Ras al Baridi		0.27819	0.29833	0.35986	0.35713
Al Wejh	0.00000 + −0.0000		0.36173	0.43717	0.49277
Oman	0.00000 + −0.0000	0.00000 + −0.0000		0.17441	0.22859
Kuwait	0.00000 + −0.0000	0.00000 + −0.0000	0.00000 + −0.0000		0.05277
Jana Island	0.00000 + −0.0000	0.00901 + −0.0091	0.00000 + −0.0000	0.03604 + −0.0148	

*Note:* The diagonal above of grey boxes denotes significance (− Not significant; +0.01 < *p* < 0.05; ++*p* < 0.01) and below are the pairwise *F*_st_ values. The coloured cells indicate the strength of significance (blue is not significant, and red is significant). Jazirat Jadir and AMAALA were omitted due to small sample size, and extended results are reported in S2.

## 4 Discussion

### 4.1 Genetic Diversity Within the Red Sea

The results presented showed population structuring among rookeries within the Red Sea, with two widespread haplotypes (CmP71.1 and CmP62.2) reported at all rookeries sampled, apart from the absence of CmP71.1 at Jazirat Jadir. The CmP71 haplogroup is unique to the Red Sea, while the CmP62.2 haplotype appears to be prevalent in the Arabian Gulf (Al‐Mohanna et al. 2014; Reece et al. 2016; Jensen, Miller, et al. 2019). The relatively low number of haplotypes identified in the Red Sea mirrors other small basins, such as the Arabian Gulf and the Mediterranean Sea (Bagda et al. 2012; Al‐Mohanna et al. 2014), with six haplotypes identified in both basins, respectively. The haplotype distribution found in the Red Sea was similar to the Arabian Gulf and the South West Indian Ocean; the Red Sea also has one dominant haplotype, CmP71.1, CmP62 in the Arabian Gulf and C3 is prevalent in the South West Indian Ocean. The CmP71.1 haplotype was found at Ras al Hadd in Oman, and CmP62 has been identified in the Arabian Gulf at sites in Kuwait and Jana Island, Saudi Arabia (Jensen, Miller, et al. 2019), but none of which have been identified at sites in the wider Indian Ocean (Bourjea et al. 2007; Phillott and Gamage 2014; Jensen, FitzSimmons, et al. 2019). Despite other known invasions of the Mediterranean Sea by Red Sea taxa (Shefer et al. 2004), there were no shared haplotypes with the Red Sea, and both these populations grouped in different phylogeographic clusters than those in the Indo‐Pacific (Bowen et al. 1992). The cosmopolitan distribution of the green sea turtle (Al‐Mansi et al. 2021; Barrios‐Garrido 2023; Mann et al. 2024) may account for the mixed haplotype distribution we observe. Although sea turtles at Ras al Baridi and Jazirat Mashabah are known to consistently return to the same nesting site throughout the nesting season (Shimada et al. 2021), it is not known how precise nest site fidelity is between nesting seasons. The unfixed nature of the identified haplotypes aligns with satellite tracking data on green sea turtles in the Red Sea (Mancini et al. 2018; Al‐Mansi et al. 2021; Shimada et al. 2021; Tanabe et al. 2023; Mann et al. 2024). Diverse post‐nesting migrations may explain population overlaps and the widespread prevalence of the haplotypes detected. However, pairwise analysis revealed significant genetic differentiation between Ras al Baridi and the rookeries in Al Wejh lagoon (Jazirat Mashabah and Jazirat Waqqadi). This differentiation is likely driven by the additional CmP71.4 haplotype at Ras al Baridi, which is absent in the Al Wejh rookeries. These findings suggest limited gene flow between the two sites, with important implications for conservation management, indicating that the populations may require distinct management strategies.

Among the rookeries surveyed, the greatest haplotype diversity was observed at Ras al Baridi, which supports 250–300 nesting females per year (Shimada et al. 2021). The greater sampling efforts and larger rookery size may be associated with higher genetic diversity, with more individuals contributing to a gene pool and reduced inbreeding (Maurer et al. 2021). The Jazirat Waqqadi rookery is markedly smaller than Jazirat Mashabah and Ras al Baridi yet maintains comparable haplotype diversity. Increased haplotype diversity is often reported at smaller rookeries due to the combined effects of immigration and imperfect homing behaviour (Formia et al. 2006; Bourjea et al. 2007), which may also be the case for this site. This is plausible for Jazirat Mashabah, near (< 200 km) Ras al Baridi and Jazirat Waqqadi, with immigration impacting smaller rookeries more. However, it seems more likely that these smaller rookeries are the result of the fragmentation of a larger ancestral population (Lahanas et al. 1994). Jazirat Mashabah and Jazirat Waqqadi are part of the Al Wejh lagoon system, a network of islands with sea turtle nesting (Shimada et al. 2021), potentially contributing to one larger rookery and may also account for an increased number of haplotypes at this site.

The rookeries sampled here reported overall similar levels of haplotype diversity compared to other rookeries within the North West Indian Ocean (Reece et al. 2016; Jensen, Miller, et al. 2019) but slightly lower than those in the Atlantic (*Hd* = 0.88; Encalada et al. 1996) and the Pacific (*Hd* = 0.88; Dethmers et al. 2006). However, in our study, we observed much higher nucleotide diversity than sites in the Atlantic (Formia et al. 2006, 2007), despite our small sample size. Estimates in this study were consistent with those elsewhere in the region (Reece et al. 2016; Jensen, Miller, et al. 2019) due to the presence of two highly divergent haplogroups at most rookeries. At a population level, high nucleotide diversity implies a high level of genetic variation among individuals, which may enhance a population's adaptability and resilience to environmental changes or diseases. The lower haplotype diversity may suggest this population recently underwent an expansion or bottleneck (Jensen, Miller, et al. 2019), and only a few haplotypes have persisted across generations. This has been observed in other Red Sea taxa, *Tridacna squamosina* (Lim et al. 2021) and sea turtle populations inhabiting the Arabian Gulf (Natoli et al. 2017). The high nucleotide diversity at Jazirat Jadir could be a result of the presence of both divergent haplogroups from the Arabian Gulf and the Red Sea, although the small sample numbers limit the strength of this inference.

While we acknowledge the sampling bias among rookeries, there is a noticeable decrease in the prevalence of the CmP71.1 haplotype and an increase in the CmP62.2 haplotype in the Southern Red Sea, as observed at Jazirat Jadir. The North and South proportions of the Red Sea are classified into two separate ecoregions (ecoregions 87 and 88) (Spalding et al. 2007). The continuity of these two ecoregions is limited by opposing cyclonic and anticyclonic eddies (Raitsos et al. 2013; Zarokanellos et al. 2017; Berumen et al. 2019), oceanographic features previously postulated to act as a barrier to gene flow in the Western Indian Ocean (Gaither et al. 2010) and in Red Sea clownfish populations (Nanninga et al. 2014). Jazirat Jadir is situated at 19° latitude, immediately south of the 20° ecoregion boundary, where there is a marked increase in chlorophyll‐a. Primary productivity has been shown to influence genetic structure in other sea turtle populations (Rodríguez‐Zárate et al. 2018). The increased presence of the CmP62.2 haplotype may reflect this, providing further evidence of this ecoregion distinction, but more sampling is needed to confirm this. However, we observed the continuity of haplotype CmP71.4 across this ecoregion boundary, and the lack of significant difference in pairwise analysis suggests gene flow exists between Ras al Baridi and Jazirat Jadir, slightly weakening this hypothesis. These findings underscore the potential role of oceanographic and ecological boundaries in shaping the genetic structure of the green sea turtle within the Red Sea, emphasising the importance of regional‐level conservation management.

### 4.2 Isolation of the Red Sea

The high levels of biodiversity and endemism observed in the Red Sea are attributed to its unique climate and past paleogeological events, such as the spreading of the seafloor to create the basin and eustatic sea‐level fluctuations (DiBattista et al. 2016). Our study identified two divergent haplotypes in our sample. The CmP71 haplogroup is separated by 19 nucleotide substitutions from the CmP62.2 haplotype. The dominance of one highly divergent haplogroup in our population may suggest two colonisation events. We hypothesise this could be due to geographic isolation following the re‐establishment of marine conditions approximately 2–5 million years ago (Girdler and Styles 1974; DiBattista et al. 2016). For instance, Jensen, FitzSimmons, et al. (2019) showed that when examining the phylogenetic relationships among all green sea turtle haplogroups, the most recent common ancestor of the Pacific Clade diverged during the late Pliocene to early Pleistocene. This divergence split into Clade XI (which includes CmP62) and other haplogroups – specifically, Clades V–X (which include, among others, CmP71) – a period that coincides with sea‐level fluctuations in the Bab‐el‐Mandeb Strait (DiBattista et al. 2016). This is in stark contrast to less isolated rookeries like Ras al Hadd in Oman, which show evidence of genetic influx from multiple basins (the Arabian Sea, the Gulf of Aden and the wider Indian Ocean). This geographic position and multiple colonisation events give rise to higher nucleotide diversity and the five haplotypes present in this rookery alone (Reece et al. 2016; Jensen, Miller, et al. 2019). The Red Sea has remained relatively isolated from the Indian Ocean, with the connectivity fluctuating over time with changes in sea level (Braithwaite 1987; Rohling et al. 1998), reinforcing barriers and increasing genetic divergence while lowering genetic diversity.

In the absence of physical barriers, genetic structure may be underpinned by environmental conditions or oceanographic currents (Jensen et al. 2020). The Red Sea is characterised by high sea surface temperature and high salinity (Chaidez et al. 2017), producing a strong environmental gradient between the Red Sea and the Indian Ocean. Climatic forcing may strengthen patterns of genetic divergence already found in other taxa (Floeter et al. 2007). Thermal profiles seem to exert a strong effect on the structuring of mtDNA in sea turtles (Bowen and Karl 2007); though analysis using multiple loci is needed to validate these patterns of isolation by environment (IBE). More recently, environmental discontinuities are thought to play a greater role in shaping genetic structure in sea turtles, as the mating pool may be limited to those adapted to specific environmental conditions (Rodríguez‐Zárate et al. 2018). The Red Sea haplogroup from this study may reflect an adaptation to elevated temperature profiles within the basin, which is of particular interest considering current warming rates. Efforts to conserve haplogroups with adaptive potential will be essential to conservation planning to maintain population resilience. Our results also support distinct regional management actions for the Red Sea green sea turtle population, given the prevalence of one highly divergent haplogroup and the unique environmental conditions influencing this population within the basin.

Satellite tracking data suggests that green sea turtles nesting in the Southern Red Sea typically remain within the basin (Mann et al. 2024; Tanabe et al. 2023). However, the large spatial distribution of this species means that dynamic interpopulation exchange between the Red Sea and the wider Indian Ocean cannot be ruled out. Previous studies have shown genetic exchange between the Red Sea and Omani rookeries (Jensen, Miller, et al. 2019), but this is the first study to demonstrate historic population connectivity between the Red Sea and the Arabian Gulf. This is likely due to the inflow of intermediate water from the Gulf of Aden toward the Southern Red Sea rookeries during the summer (Yao et al. 2014). Additionally, infrequent long‐distance colonisation events may occur, as shown by a turtle tagged in Oman entering the Southern Red Sea (Pilcher et al. 2021). Additional sampling of Gulf of Aden rookeries would provide a more comprehensive overview of genetic structure around the Arabian Peninsula and may better explain the presence of the CmP62.2 haplotype in the Red Sea.

### 4.3 Future Directions

Given that sea turtles are ectothermic animals and exhibit temperature‐dependent sex determination (TSD), increased efforts are required to fully comprehend their thermal preference amidst anthropogenic climate change. As populations become increasingly feminised (Jensen et al. 2018; Tanabe et al. 2020), there is a potential for a loss in genetic diversity due to inbreeding effects, which will require high‐resolution, genome‐wide markers for robust estimation. Conserving rookeries such as Jazirat Jadir to strengthen connectivity between the Arabian Gulf and Red Sea will be crucial to preserving both haplogroups and increasing overall genetic diversity. Further, the mixing of two divergent haplogroups creates ‘genetic hotspots’ and areas potentially more resilient to climate change and provides refuge for future climatic events.

Genome‐wide markers offer the potential to overcome the limitations of single‐locus, maternally inherited markers like mtDNA in delineating populations and estimating their demographic histories. For instance, coalescent‐based models could be examined based on these high‐resolution markers to more precisely determine the migration patterns and divergence times of Red Sea haplogroups compared to other Pacific green turtle lineages (Van Der Zee et al. 2021). Genome‐wide association studies could also be used to locate genes selected for in Red Sea populations that are better adapted to persist at elevated temperatures, which will result in the identification of more resistant genetic stocks. Additional sampling, particularly of underrepresented Southern Red Sea rookeries and sampling in Yemen, would enhance our understanding of genetic connectivity around the Arabian Peninsula, and coupling genetic studies with other approaches, such as hatchling dispersal models, could provide better insights into dispersal pathways and genetic flow in the region.

## 5 Conclusions

This study is the most comprehensive categorisation of breeding stocks among multiple green sea turtle rookeries in the Red Sea, increasing the sample size 10‐fold. We have shown strong population differentiation between the Red Sea and the Indian Ocean and detected four additional haplotypes not previously identified in this basin. The dominance and deep divergence of the CmP71 haplogroup suggest that physical and environmental barriers in the Red Sea are the primary drivers behind the patterns observed. Conservation of this haplogroup may be essential to the resilience of the Red Sea population, and its evolutionary potential warrants further study. Finally, this was the first study to show the CmP62 haplotype, dominant in the Arabian Gulf within the Red Sea, demonstrating further current or historical connectivity in green sea turtle populations between these basins.

## Author Contributions

**K. Scott:** conceptualization (equal), data curation (equal), formal analysis (lead), investigation (lead), methodology (lead), writing – original draft (lead), writing – review and editing (equal). **S. He:** formal analysis (lead), methodology (equal), visualization (lead), writing – review and editing (equal). **R. S. Hardenstine:** data curation (equal), investigation (equal), supervision (lead), writing – review and editing (equal). **L. K. Tanabe:** data curation (equal), investigation (equal), methodology (equal), writing – review and editing (equal). **H. Barrios‐Garrido:** data curation (equal), methodology (equal), writing – review and editing (equal). **M. P. Jensen:** conceptualization (equal), formal analysis (equal), methodology (equal), supervision (equal), validation (lead), writing – review and editing (equal). **L. Afiq‐Rosli:** formal analysis (equal), methodology (equal), writing – review and editing (equal). **N. E. Wildermann:** conceptualization (equal), data curation (equal), project administration (lead), writing – review and editing (equal). **C. M. Duarte:** conceptualization (equal), funding acquisition (lead), resources (equal), writing – review and editing (equal). **J. E. M. Cochran:** conceptualization (equal), supervision (lead), writing – review and editing (lead). **M. L. Berumen:** conceptualization (equal), funding acquisition (lead), supervision (lead), writing – original draft (equal), writing – review and editing (equal).

## Conflicts of Interest

The authors declare no conflicts of interest.

## Supporting information


**Data S1:** ece372046‐sup‐0001‐DataS1.xlsx.

**Data S2:** ece372046‐sup‐0002‐DataS2.docx.

## Data Availability

Metadata on all samples can be downloaded here file name: Sea_Turtle_Genetic_Samples_metadata.xls. Sequencing results are available in GenBank (see Section 2).
