# Supplementary material for: Genetic Divergence and Isolation of the Green Sea Turtle (Chelonia mydas) in the Red Sea
Source: Ecol Evol. 2025 Aug 25;15(8):e72046. doi: 10.1002/ece3.72046 (PMC12378018; doi:10.1002/ece3.72046)
Supplement: Supplementary file 2 — Data S2: ece372046‐sup‐0002‐DataS2.docx. [file ECE3-15-e72046-s001.docx]

**S1** Haplotype network for the green turtle populations based on 710 bp mitochondrial control region fragments, distributed in the Red Sea and 384 bp of the Arabian Gulf (Al‐Mohanna et al., 2014; Reece et al., 2016; Jensen, et al., 2019b; Mobaraki et al., 2020), with only substitutions considered. The legend indicates where distinct rookery colours are found the haplotype, and the size of the circles is proportional to their total frequency. Each branch connecting different circles represents the mutation steps among the haplotypes, with the black crossbars representing an additional nucleotide substitution, a single deletion/insertion, and the double bars representing greater than one nucleotide substitution. Unknown samples are those with unknown natal origin.

**S2** Pairwise *F_st_* values calculated using 10,100 permutations between nine *Chelonia mydas* rookeries on the Arabian Peninsula (this study, (Al‐Mohanna et al., 2014; Jensen, Miller, et al., 2019; Mobaraki et al., 2020; Reece et al., 2016), above the diagonal of grey boxes are significant and below are the pairwise *F_st_* values. Not significant; + 0.01 < *p* < 0.05; ++ *p* < 0.01, Corrected for multiple comparison, (Narum, 2006).

| AMAALA | Jazirat Mashabah | Jazirat Waqqadi | Ras al Baridi | Jazirat Jadir | Kuwait | Jana Island | Iran | Oman | **Rookery** |
| --- | --- | --- | --- | --- | --- | --- | --- | --- | --- |
|  | - | - | - | - | ++ | ++ | ++ | ++ | **AMAALA**  (n = 5) |
| -0.10221 |  | - | - | + | ++ | ++ | ++ | ++ | **Jazirat Mashabah** (n = 32) |
| -0.15744 | -0.01283 |  | - | - | ++ | ++ | ++ | ++ | **Jazirat Waqqadi**  (n = 13) |
| -0.0759 | -0.01906 | 0.01704 |  | ++ | ++ | ++ | ++ | ++ | **Ras al Baridi**  (n =160) |
| 0.31416 | 0.5871 | 0.33236 | 0.63703 |  | - | - | + | - | **Jazirat**  **Jadir**  (n = 4) |
| 0.25059 | 0.40405 | 0.27931 | 0.52815 | 0.08564 |  | ++ | ++ | - | **Kuwait**  (n = 95) |
| 0.33832 | 0.46271 | 0.35167 | 0.5631 | 0.03121 | 0.14946 |  | - | ++ | **Jana Island**  (n =11) |
| 0.375 | 0.599 | 0.425 | 0.689 | 0.207 | 0.198 | 0.053 |  | + | **Iran**  (n = 53) |
| 0.2378 | 0.4032 | 0.2681 | 0.5256 | 0.0928 | 0.0017 | 0.1744 | 0.2286 |  | **Oman**  (n =42) |
